# Supplementary material for: Investigation of Antibacterial and Antiinflammatory Activities of Proanthocyanidins from Pelargonium sidoides DC Root Extract
Source: Nutrients. 2019 Nov 19;11(11):2829. doi: 10.3390/nu11112829 (PMC6893413; doi:10.3390/nu11112829)
Supplement: Supplementary file 1 [file nutrients-11-02829-s001.pdf]

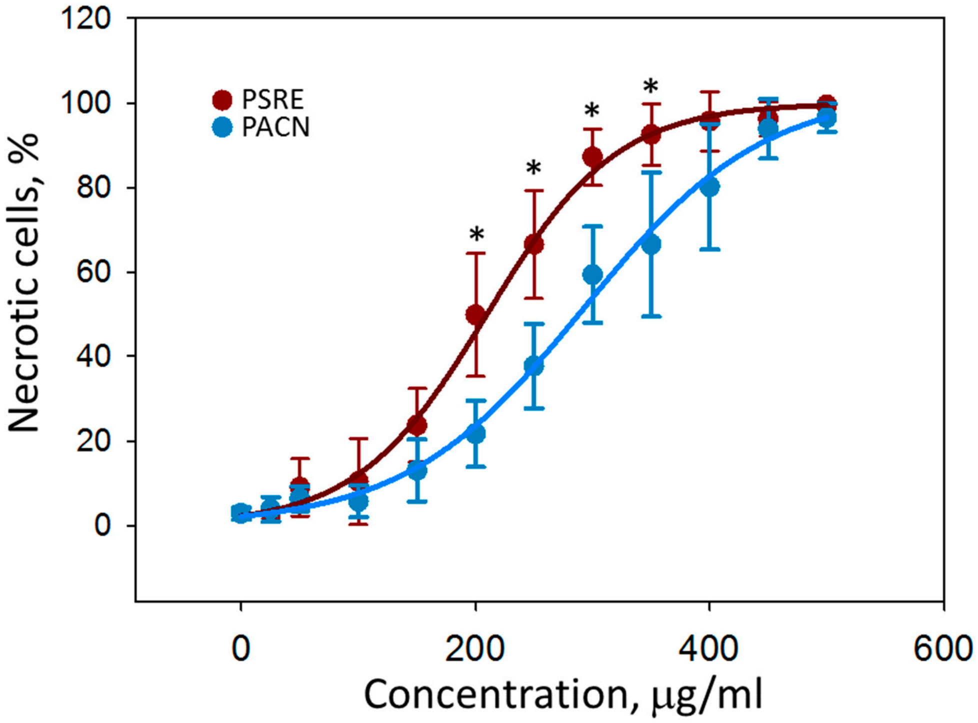


**Supplementary Figure S1.** The concentration-dependent toxicity of *Pelargonium* sidoides DC root extract (PSRE) and proanthocyanidins from PSRE (PACN) for rat gingival fibroblasts. The cells were incubated with the extracts at different concentrations for 24 h period and necrotic vs total cell assessment was performed by double nuclear staining (propidium iodide and Hoeachst3334) and fluorescent microscope. Data are presented as means of 5 experimental repeat data plus standard deviation, and curve fitting analysis was done by SigmaPlot vs.13 software. * - significant difference compared to the samples treated with the same concentration of PACN ($p<0.05$).

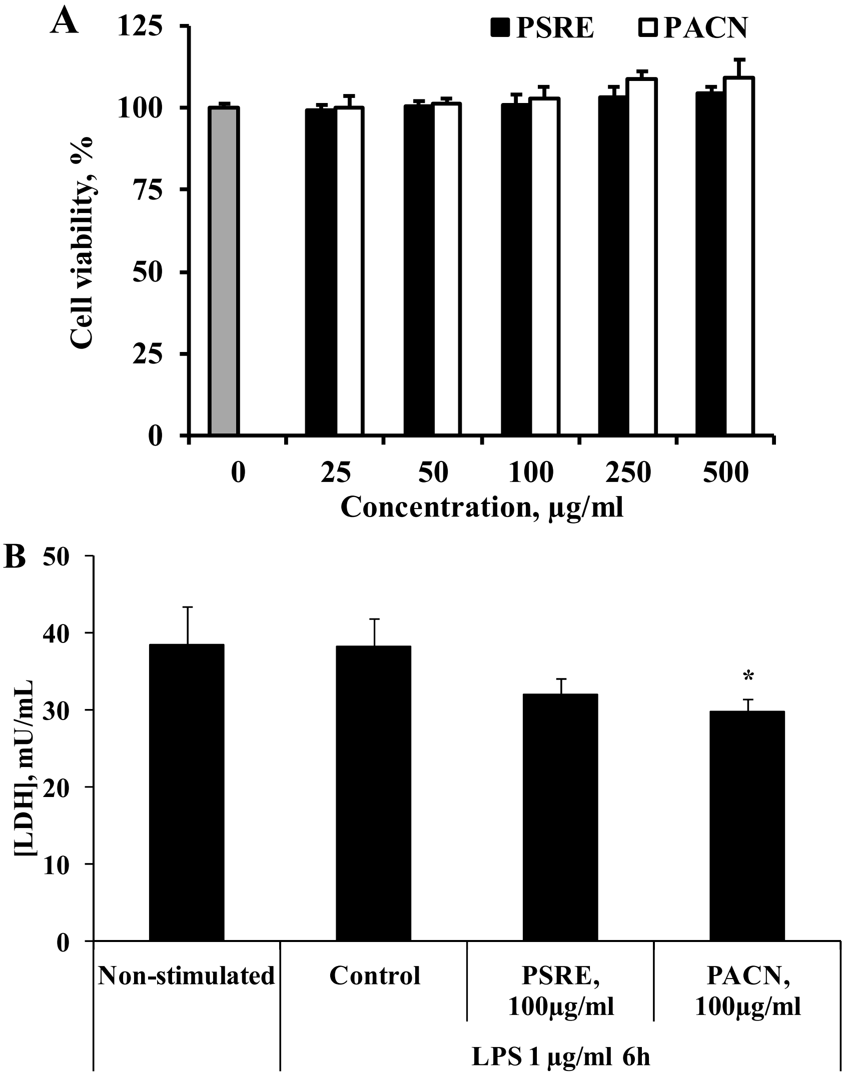


**Supplementary Figure S2.** Effects of *Pelargonium sidoides* DC root extract (PSRE) and proanthocyanidins from PSRE (PACN) on human peripheral blood mononuclear cell viability. (A) Human peripheral blood mononuclear cell viability after 24h incubation with PSRE and PACN evaluated by Alamar Blue assay. (B) Effects of PSRE and PACN on blood mononuclear cell membrane damage measured by lactate dehydrogenase (LDH) assay. The LDH release was tested in media after 6 h treatment with extracts (100 µg/mL) and LPS (1 µg/mL). Values are represented as the mean ± SD of (A) 6 parallels or (B) 3 independent measurements in 3 parallels. Differences between the measurements were tested using one-way ANOVA followed by Tukey's Multiple Comparison Test. * - significant difference compared to the LPS control ($p < 0.05$).

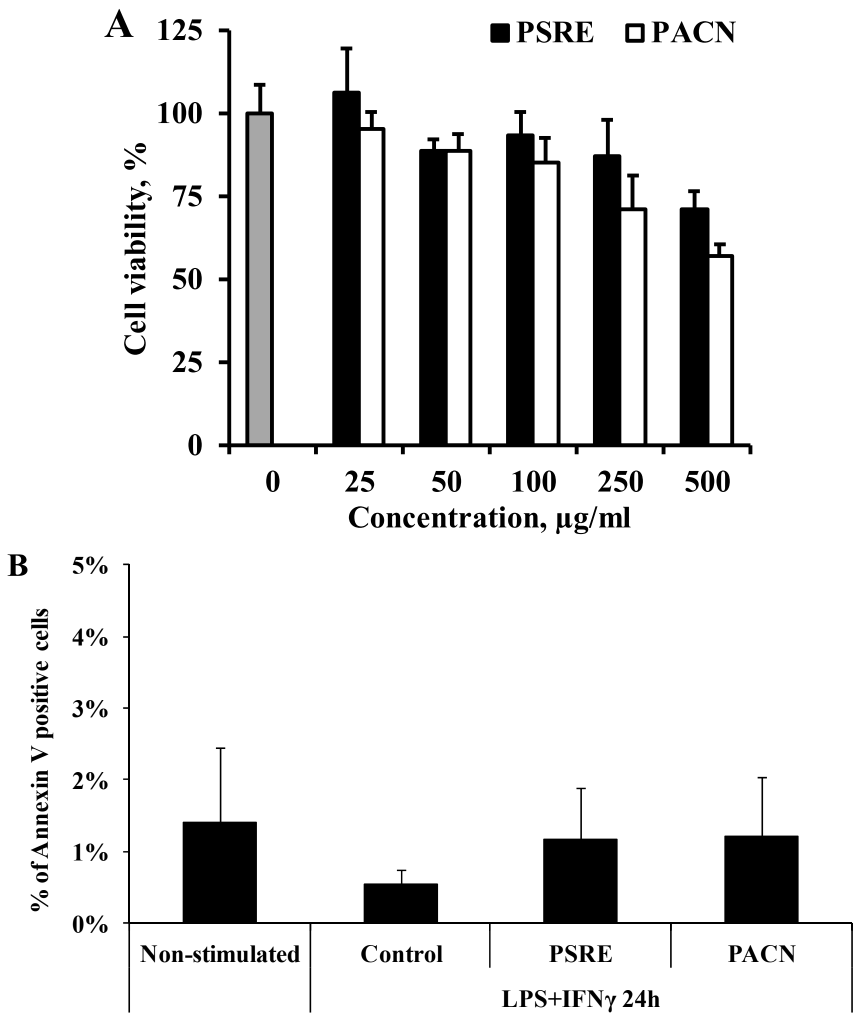


**Supplementary Figure S3**. The effect of *Pelargonium sidoides* DC root extract (PSRE) and proanthocyanidins from PSRE (PACN) on Detection of apoptosis by staining bone-marrow derived macrophages (BMDM). (A) BMDM viability after 24h incubation with PSRE and PACN assessed by MTT assay. (B) Detection of apoptosis by staining BMDM for Annexin V. BMDM were stained for Annexin V after 24 h of incubation with PSRE and PACN at 100 µg/mL, and LPS+IFN-γ (10 ng/mL/100 U/mL). Apoptotic cells (Annexin V positive) were detected by flow cytometry. Values are presented as mean ± SD of (A) 6 parallels or (B) 3 independent measurements in 3 parallels.
